# Mercury Removal by Carbon Materials with Emphasis on the SO_2_–Porosity Relationship

**DOI:** 10.1002/open.202500190

**Published:** 2025-07-15

**Authors:** Maria Antonia López‐Antón, Lucia López‐Toyos, Sara F. Villanueva, Elena Rodríguez, Roberto García, Maria Rosa Martínez‐Tarazona, Ana Arenillas

**Affiliations:** ^1^ Instituto de Ciencia y Tecnología del Carbono INCAR‐CSIC C/ Francisco Pintado Fe, 26 33011 Oviedo Spain

**Keywords:** elemental mercury, flue gases, iron oxides, pore structures, SO_2_ resistance

## Abstract

Mercury is a pollutant of great global concern. Although numerous studies have been carried out for its removal from energy production processes, there are still some gaps in this field that must be filled to improve the development of adsorbents/catalysts capable of retaining it. In this study, a model material with controlled pore structure is developed to evaluate the effect of pore structure on SO_2_ tolerance during Hg^0^ adsorption. The carbon material is loaded with different active species of iron. The results show that hematite is the reactive iron species for Hg capture. In contrast to the general assumption, a well‐developed microporosity is not the only textural parameter that should be considered to improve flue gas Hg retention. In fact, highly microporous materials are prone to SO_2_ poisoning. Therefore, the role of porosity in mercury capture in the presence of SO_2_ must be evaluated from a new perspective, taking into account the textural characteristics as a whole. The developed model demonstrates that a carbonized material can be as effective for mercury removal as a more expensive activated carbon material, responding to the growing demand for cost‐effective technologies.

## Graphical abstract

The effect of textural properties on the Hg/SO_2_ relationship is key to developing cost‐effective technologies for mercury removal in energy production processes. Furthermore, the developed model material, with a hierarchically controlled pore structure, provides new insights that can be applied to other carbon‐based adsorbents/catalysts in different applications.

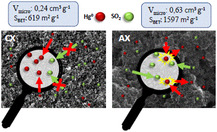

## 1 Introduction

The World Health Organization (WHO) states that exposure to Hg produces harmful effects on the nervous, digestive, and immune systems, as well as on the lungs and kidneys, all of which may be fatal.^[^ ^1^ ^]^ The incessant emission from different anthropogenic sources has caused a ubiquitous presence in the environment that has tripled in the last few decades. Hence, the need to reduce mercury emissions and releases into land and water is a matter of urgency. In this regard, different agreements and regulations, such as the Minamata Convention on Mercury, a directive of the European Parliament, the US Environmental Protection Agency, and China's Emission Standard of Air Pollutant for Thermal Power Plants (GB13223‐2011), have been established.^[^ ^2^, ^3^, ^4^, ^5^ ^]^

The combustion of fossil fuels for energy production is one of the primary anthropogenic sources of mercury emissions (accounting for ≈21% of emissions to the air). Although the demand for coal for energy production has decreased in recent years in European countries, it has increased in Asian countries.^[^ ^6^, ^7^ ^]^ Therefore, as reflected in various international programs,^[^ ^8^ ^]^ controlling mercury emissions from this sector remains a global challenge that must be addressed.

As stated in the literature, with numerous reviews conducted,^[^ ^9^, ^10^, ^11^, ^12^, ^13^, ^14^, ^15^ ^]^ various technologies have been developed to control mercury in coal combustion flue gas. Most methods are actually control technologies designed for other pollutants, such as particulate matter collection units (e.g., electrostatic precipitators or fabric filters), selective catalytic reduction systems for NOx control, and desulfurization units, all of which exhibit a synergistic effect on mercury removal. The only specific technology for controlling elemental mercury (Hg^0^) is the injection of adsorbents. It must be noted that Hg^0^ is the most challenging species to capture due to its high volatility and low solubility. This makes its retention particularly difficult compared to other mercury species. The aforementioned adsorption onto solid materials, as well as catalytic oxidation, are the technologies currently gaining the most attention for addressing Hg^0^ removal.^[^ ^5^, ^16^, ^17^, ^18^, ^19^, ^20^ ^]^

There are numerous studies focused on the development of adsorbents and catalysts for the retention of Hg^0^ from coal‐fired power plants.^[^ ^21^, ^22^, ^23^, ^24^, ^25^ ^]^ Most adsorbents are modified to improve the surface pore structure and/or increase the concentration of active centers. Those modified with halides (iodine, bromine, and chlorine) and sulfur have been widely studied and have been demonstrated to have strong chemical activity for Hg^0^, with almost 100% retention efficiency.^[^ ^26^ ^]^ However, one of the drawbacks of this type of adsorbent is their regeneration and recovery, which can lead to a new source of mercury‐contaminated waste. Another relevant group of adsorbents includes those doped with metals (Pd, Pt, Ag, Au, etc.) and metal oxides (MnOx, CuOx, FexOy, etc.), which not only effectively catalyze Hg^0^ oxidation in flue gases but can also be easily regenerated.^[^ ^24^, ^27^ ^]^ In particular, adsorbents based on iron oxides have been widely used due to their high efficiency, low cost, and the potential for regeneration.^[^ ^28^, ^29^, ^30^ ^]^

On the other hand, it is well known that, among other characteristics (temperature, flow rate, etc.), the composition of flue gas significantly affects the activity of adsorbents and catalysts, and numerous studies have been conducted on this matter.^[^ ^14^, ^31^ ^]^ SO_2_ is one of the flue gas components most commonly evaluated for its inhibitory effect on Hg^0^ removal, acting through the following primary mechanisms: 1) competitive adsorption, 2) consumption of active sites, and 3) formation of sulfates that poison the adsorbent/catalyst.^[^ ^16^, ^32^, ^33^ ^]^ Although various strategies can be implemented to mitigate the inhibitory effect of SO_2_, such as increasing the number of active sites and suppressing SO_2_ adsorption,^[^ ^32^ ^]^ the development of a pore structure optimized to enhance SO_2_ tolerance has not yet been thoroughly evaluated. The impact on SO_2_ resistance of the combination of microporosity, mesoporosity, and macroporosity in the texture of adsorbents/catalysts is difficult to assess, as developing materials with a hierarchically controlled pore structure is typically not an easy task.

In this work, a model material was developed to further assess the role of pore structure in SO_2_ resistance during Hg^0^ adsorption in coal combustion processes. The goal was not to create a new material for mercury removal but rather to address knowledge gaps in this area from a textural characterization perspective, enabling the application of these insights to other carbon‐based adsorbents/catalysts.

## 2 Results and Discussion

In this study, the results are discussed based on: 1) the retention of mercury by the raw and Fe‐loaded carbon materials in a simulated flue gas and 2) the influence of textural characteristics on mercury retention capacity in the presence of SO_2_.

### 2.1 Physicochemical Characterization of the Materials Tested

The amount of Fe supported was not quantitative for the organic xerogel (OX) and carbonized material (CX) samples (6–7 wt%) (**Table** **1**). However, for the activated materials (AX), the impregnation efficiency reached up to 85% (17 wt%). Generally, a higher surface area promotes a more quantitative immobilization of Fe on the support (Table 1). However, the surface chemistry of the samples may also influence their interaction with Fe during the immobilization process. Therefore, although the Fe content in the OX and CX samples is similar (6–7 wt%), their performance can be compared in terms of their differing textural properties.

**Table 1:** Textural properties and iron concentration of the carbon materials.

	Fe	*S*_BET_	*S*_ext_	*V*_micro_	*V*_t_	*ρ*_He_
	[wt%]	[m^2^ g^−1^]	[m^2^ g^−1^]	[cm^3^ g^−1^]	[cm^3^ g^−1^]	[g cm^−3^]
OX	–	226	202	0.11	0.41	1.40
CX	–	619	145	0.24	0.38	1.90
AX	–	1597	509	0.63	1.03	2.00
OXFe(g)	6.30	158	143	0.06	0.27	1.44
OXFe(h)		72	66	0.03	0.25	3.04
CXFe(g)	6.84	523	142	0.21	0.34	1.98
CXFe(h)		585	170	0.23	0.36	1.88
AXFe(g)	17.1	1325	442	0.51	0.89	1.93
AXFe(h)		1462	601	0.58	0.98	2.29

The sol‐gel methodology enables the design of mesopores (i.e., pores between 2 and 50 nm in width), often referred to as feeder pores, during the sol‐gel process, while microporosity (i.e., pores <2 nm in width) can be developed independently during postsynthesis treatments. Consequently, the OX, CX, and AX samples exhibit similar mesoporosity, with pores in the 6‐8 nm range (Figure S1a, Supporting Information), but they show significant differences in microporosity, as indicated by the volume of N_2_ adsorbed at low relative pressures (Figure S1b, Supporting Information).

All the supports exhibit type I–IV isotherms according to the IUPAC classification,^[^ ^34^ ^]^ which are characteristic of micro‐ and mesoporous samples with a well‐defined capillary condensation step at p/p0 above 0.5, which is indicative of a well‐developed mesoporosity, consistent with uniform cylindrical mesopores and greater pore connectivity. This is confirmed by the pore size distribution (PSD), which shows that the porosity is made up of uniform mesopores ranging from 2 to 10 nm. The presence of a hysteresis loop also suggests a similar mesoporous structure across the samples. The carbonization process (i.e., heat treatment under an inert atmosphere) removes volatile matter in CX, resulting in the development of microporosity, which is reflected in higher adsorbed volumes at low p/p^0^ compared to the original polymer OX. The activation process used during the synthesis of AX further enhances this microporosity, as indicated by the increased adsorption at low relative pressures in Figure S2b, Supporting Information. This increase in microporosity contributes to a higher specific surface area (*S*_BET_), as summarized in Table 1, following the trend OX < CX < AX (226, 619, and 1597 m^2^ g^−1^, respectively).

Incorporation of Fe into these samples partially blocks the pores, leading to a decrease in *S*_BET_ compared to the pristine samples: 158, 523, and 1325 m^2^ g^−1^ for OXFe(g), CXFe(g), and AXFe(g), respectively. A slight increase in the *S*_BET_ is observed when these samples undergo heat treatment to convert goethite to hematite, as seen in CXFe(h) and AXFe(h). However, this increase is not observed for OXFe(h), where a notable change in morphology occurs. The low thermal stability of the OX sample may induce secondary reactions with the Fe precursors, further blocking the porosity, as indicated by the reduction in both microporosity and *S*_BET_. Additionally, the N_2_ adsorption–desorption isotherm shifts to type II (Figure S2, Supporting Information), indicating the loss of mesopores. Accordingly, OXFe(h) is completely different from OXFe(g), not only in terms of its chemistry but also in porosity of the resultant material, with a denser structure clearly reflected by the increase of helium density and the decrease of the porous characteristics (Table 1).

**Figure 1:**
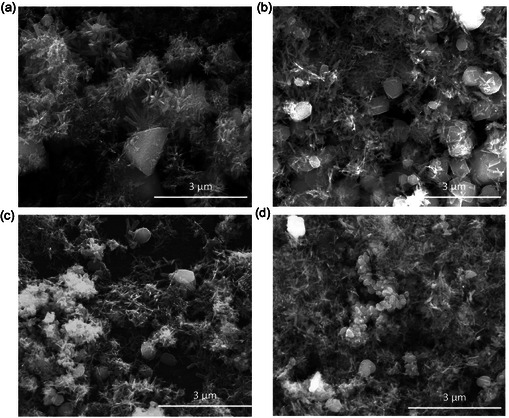
SEM images of a) OXFe(h), b) CXFe(h), c) AXFe(h), and d) AXFe(g).

The morphologies of Fe‐impregnated samples were examined using scanning electron microscopy (SEM) (**Figure** **1**). Iron species predominantly appeared as polyhedra and nanorods. However, the distribution of these iron species was more uniform in the CXFe and AXFe samples compared to OXFe (Figure 1a–c). For the OXFe material, a lower surface coverage was observed, with noticeable agglomeration of nanorods forming in certain areas (Figure 1a).

The analysis by X‐ray diffraction (XRD) (Figure S3a, Supporting Information) revealed the presence of goethite in the samples synthesized by oxidative hydrolysis (OXFe(g), CXFe(g), and AXFe(g)). The subsequent heat treatment at 300 °C led to the transformation of goethite into hematite (OXFe(h), CXFe(h), and AXFe(h)) (Figure S3b, Supporting Information). Notably, the original polyhedral and nanorod morphologies of the iron species were preserved after this thermal transformation (Figure 1c,d).

As observed in previous studies conducted by the authors,^[^ ^35^ ^]^ the type of iron species present on the surface of carbon materials significantly impacts mercury adsorption performance. This performance is also influenced by the textural properties of the material and, distinctly, by the presence of SO_2_.

### 2.2 Mercury Retention: The Role of Porous Texture

**Figure 2:**
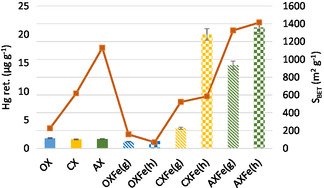
Mercury retention capacity and surface area of carbon materials. Flue gas composition: 100 μg m^−3^ Hg^0^, 15% CO_2_, 6% O_2_, 8% H_2_O, 100 ppm SO_2_, balance N_2_. Temperature: 80 °C.

**Figure** **2** shows the mercury retention capacity alongside the surface area for all the carbon materials studied. As previously discussed, these materials exhibit variable textural characteristics but were designed with hierarchical porosity to enable a comprehensive evaluation of their effects on mercury removal and SO_2_ resistance. It is worth noting that OX and CX samples have comparable total pore volumes and mesopore sizes but differ in micropore volume, leading to differences in *S*_BET_ (Table 1). In contrast, the AX samples have a more developed porosity with higher pore volumes across the entire porosity range. The mercury retention capacity varies according to the type of carbon material and the iron species present.

Similar mercury retention capacities (1–2 μg g^−1^) were observed in the raw materials and the OX sample loaded with iron species (OXFe(g) and OXFe(h)), regardless of their textural properties (Figure 2, Table 1). This suggests that the carbonaceous matrix itself lacks significant activity for mercury capture and serves mainly to provide a support with appropriate porosity for the active phase. Additionally, the limited textural properties of the OXFe samples reduce the accessibility of Fe active sites, leading to low mercury removal activity irrespective of the type of iron species present.

Impregnation of the CX and AX with Fe had a significant impact on Hg capture efficiency, with the highest retention observed in CX and AX samples containing hematite (20 and 21 μg g^−1^ for CXFe(h) and AXFe(h), respectively). Previous studies have shown that the outer layer of Fe^3+^ in the iron oxide (αFe_2_O_3_), which has an empty orbital structure, enhances the Fe/Hg^0^ interaction .^[^ ^36^ ^]^ In fact, earlier research by the authors on carbon foams demonstrated that αFe_2_O_3_ enhances mercury capture efficiency compared to other iron oxides and hydroxides under similar conditions.^[^ ^35^ ^]^ The results obtained in this work corroborate these findings, as both CXFe(h) and AXFe(h) exhibit higher mercury retention values than CXFe(g) and AXFe(g), respectively, reinforcing the role of αFe_2_O_3_ in Hg removal.

In terms of textural properties, it is notable that CXFe(h) and AXFe(h) exhibit similar mercury retention capacities, despite CXFe(h) having a considerably smaller surface area than AXFe(h) (Table 1, Figure 2). In contrast, for samples loaded with goethite (CXFe(g) and AXFe(g)), mercury retention is higher in AXFe(g) than in CXFe(g). Both samples have the same iron morphology, characterized by nanorods and polyhedral particles (Figure 1), but AXFe(g) benefits from a more developed microporosity, which likely enhances the availability of active sites and thus contributes to its superior Hg retention.

The results suggest that 1) the formation of αFe_2_O_3_ on the surface of carbon materials creates active sites for the chemisorption/oxidation of elemental mercury, but the critical factor is the overall porosity of the material, rather than solely the microporosity, as commonly suggested in most studies carried out so far; and 2) when FeOOH is the iron species, which results in fewer active sites available for Hg^0^ oxidation,^[^ ^36^ ^]^ a more developed pore structure with a higher surface area in AXFe(g) compared to CXFe(g) (Table 1) enhances mercury retention (Figure 2). The findings also indicate that a carbonization process alone may suffice, eliminating the need for activation and thus potentially lowering the cost of this technology.

### 2.3 Mercury Retention: The Role of SO_2_

**Figure 3:**
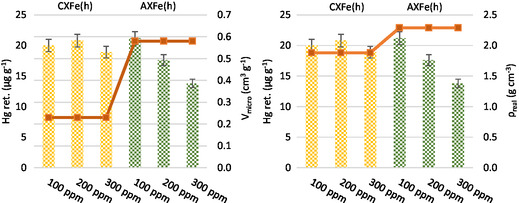
Mercury retention capacity of CXFe(h) and AXFe(h) under varying SO_2_ concentrations, illustrating the effect of differing textural properties. Flue gas composition: 100 μg m^−3^ Hg^0^, 15% CO_2_, 6% O_2_, 8% H_2_O, balance N_2_. Temperature: 80 °C.

Inhibition of mercury capture could occur because carbon material is a catalyst for the oxidation of SO_2_ to sulfuric acid or through adsorption of SO_3_ (which can hydrolyze to sulfuric acid), with SOx competing with mercury for the same adsorption sites, specifically the Lewis base sites, on the carbon surface. Two carbon materials with differing pore structures were subjected to varying SO_2_ concentrations to assess the effect on Hg^0^ adsorption, specifically focusing on those materials that exhibited the highest mercury retention capacity, that is, those loaded with αFe_2_O_3_ (CXFe(h) and AXFe(h)). **Figure** **3** displays the mercury retention capacity of CXFe(h) and AXFe(h) at different SO_2_ concentrations, highlighting their contrasting textural properties. For CXFe(h), mercury retention remained relatively stable across the SO_2_ concentration range. In contrast, AXFe(h), characterized by a more developed porosity, showed a progressive decrease in mercury removal as SO_2_ concentration increased (Figure 3). This suggests that SO_2_ competes with Hg^0^ for surface and lattice oxygen active sites, thereby reducing Hg^0^ adsorption in the material (M) via the well‐known Mars–Maessen mechanism.^[^ ^35^, ^37^, ^38^ ^]^

(1) Hg(g) + M → Hg(ads)

(2) $$\text{Hg}\left(\text{ads}\right){\text{ + αFe}}_{2}O_{3}\to \text{HgO}\left(\text{ads}\right)\text{ + 2FeO}$$

(3) $$\text{HgO}\left(\text{ads}\right){\text{ + 2FeO + ½ O}}_{2}\to \text{ HgO}\left(\text{ads}\right){\text{ + Fe}}_{2}O_{3}$$

(4) $$\text{HgO}\left(\text{ads}\right){\text{ + Fe}}_{2}O_{3}\to {\text{ HgFe}}_{2}O_{4}$$

It is generally assumed that the first step, namely, the physisorption process (I), is favored by a higher surface area, as seen in AXFe(h) (Table 1). However, unlike CXFe(h), a more developed microporosity preferentially promotes the adsorption of SO_2_ over Hg^0^ on the same active sites, supporting a mechanism of competitive adsorption between SO_2_ and Hg^0^^[^ ^32^ ^]^ (Figure 3). Therefore, the findings of this study demonstrate that in flue gas atmospheres, an adsorbent with increased microporosity does not necessarily achieve higher Hg retention efficiency or better tolerance to acidic gases like SO_2_, which is critical for designing a cost‐effective technology optimized for mercury capture.

Although evaluating this effect is not the primary objective of this study, as it has already been investigated in various sorbents, it is important to note that hematite active sites may be consumed during retention experiments. However, this deactivation can be counteracted by the presence of O_2_ in the gas stream, which facilitates the regeneration of the sorbents.^[^ ^29^, ^35^, ^39^ ^]^

## 3 Conclusions

The development of a model material with hierarchical porosity enabled an assessment of the impact of textural properties on both Hg capture and SO_2_ resistance, a task that has proven challenging for most materials studied to date.

The similar total pore volumes in samples OX and CX suggest that this parameter does not play a crucial role in Hg adsorption. The higher Hg removal observed with CX and AX compared to OX supports the idea that a well‐developed microporosity enhances Hg retention capacity. However, contrary to common assumptions, microporosity is not the only textural parameter to consider. This is illustrated by the similar retention capacities of CXFe(h) and AXFe(h), highlighting the importance of considering porosity as a whole. It should also be noted that sorbents with more developed porosity were more affected by the presence of SO_2_.

The comparable mercury retention capacities observed between the CX and the AX also indicate that an activation process, which is more costly and tedious, is not always required. Therefore, the findings of this study not only improve the understanding of the Hg/SO_2_/texture relationship but also contribute to the development of the best available techniques and the best environmental practices for Hg control in relevant emissions sources, such as coal combustion processes.

## 4 Experimental Section

4.1

4.1.1

##### Carbon Material Preparation and Characterization

To control the porous properties of the tested materials, a synthetic polymer was obtained via the polycondensation of resorcinol (R) and formaldehyde (F) using a microwave‐assisted sol‐gel methodology.^[^ ^40^ ^]^ R was dissolved in water under stirring, and F was added to the mixture at a molar ratio of R/F = 0.5. The mixture was stirred until a homogeneous solution was achieved. The amount of water used corresponded to a dilution ratio of 5.7. The pH of the solution was adjusted to 6.5 by adding NaOH, promoting the polymerization reaction. The precursor solution was placed in a microwave oven at 85 °C for 5 h to complete gelation, aging, and drying. The resulting polymeric material, designated as OX, was subsequently heat‐treated under an inert N_2_ atmosphere at 850 °C, removing volatile matter and yielding a thermally stable material composed primarily of carbon (>95 wt%), referred to as CX. Additionally, OX was treated under a reactive CO_2_ atmosphere at 1000 °C to introduce structural defects, which result in microporosity and surface area, also removing volatile components. This process yielded the AX used in this study.

OX, CX, and AX were impregnated with 20 wt% Fe using a solution of iron sulfate heptahydrate (FeSO_4_ · 7H_2_O) and sodium acetate (CH_3_COONa) in MilliQ water under reflux for 2 h.^[^ ^35^ ^]^ The Fe content in the materials was determined by inductively coupled plasma mass spectrometry after acid digestion of the sample in a microwave oven. A portion of the samples underwent an additional thermal treatment at 300 °C in a muffle furnace for 2 h, resulting in different phases and morphologies of iron species. The final samples were designated as OXFe(x), CXFe(x), and AXFe(x), where × indicates the predominant iron species present in the carbon material (g: goethite; h: hematite).

The distribution and morphology of the iron nanoparticles were studied using SEM, and the identification of the iron species was performed by XRD.

The textural characteristics of the tested samples were evaluated using N_2_ adsorption–desorption isotherms at −196 °C. The *S*_BET_ of the carbon materials was determined by applying the Brunauer–Emmett–Teller equation, and the external surface area was evaluated using the *t*‐method. The total pore volume (*V*_t_) was obtained from the quantity of N_2_ adsorbed near the saturation pressure (*p*/*p*_0_ = 0.99). The micropore volume (*V*_micro_) was determined using the Dubinin–Radushkevich equation. The PSD was calculated with the 2D‐NLDFT heterogeneous model. Additionally, the He density was measured for all the samples studied.

##### Mercury Experimental Device

The effect of SO_2_ concentration on mercury retention capacity was evaluated in a fixed‐bed reactor (Figure S4, Supporting Information) using SO_2_ levels of 100, 200, and 300 ppm. The simulated flue gas composition included 15% CO_2_, 6% O_2_, and 8% H_2_O, with N_2_ as the balance. Currently, sorbents and catalysts are designed to be used in power plants where DeNox, particulate control, and desulphurization devices are operating. Hg^0^ was introduced into the gas stream from a commercial permeation tube, achieving a concentration of 100 μg m^−3^. The sample was placed inside a glass reactor and maintained at 80 °C. The concentration of Hg^0^ not retained by the fixed‐bed was monitored using a VM‐3000 analyzer. Any oxidized mercury (Hg^2+^) not retained by the sorbent was captured using a Dowex 1 × 8 ion‐exchange resin, which selectively captures Hg^2+^.^[^ ^41^ ^]^ The Hg^2+^ retained in the resin was then quantified with an AMA 254 automatic mercury analyzer.

## Conflict of Interest

The authors declare no conflict of interest.

## Supporting information

Supplementary Material

## Data Availability

The data that support the findings of this study are available from the corresponding author upon reasonable request.
