# Supplementary material for: Mercury Removal by Carbon Materials with Emphasis on the SO2–Porosity Relationship
Source: ChemistryOpen. 2025 Jul 15;14(11):e202500190. doi: 10.1002/open.202500190 (PMC12598800; doi:10.1002/open.202500190)
Supplement: Supplementary file 1 — Supplementary Material [file OPEN-14-e202500190-s001.pdf]

Supporting information

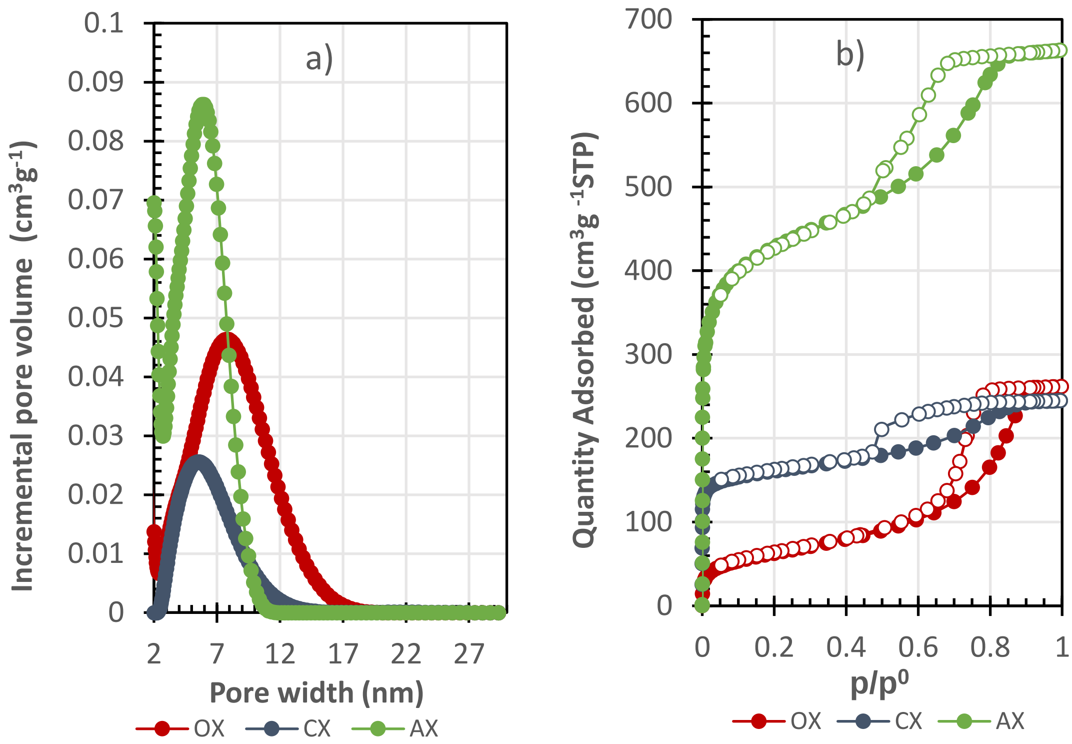

Figure S1. a) Pore size distribution and b) N_2 adsorption-desorption isotherms of the studied supports: OX, CX, and AX.

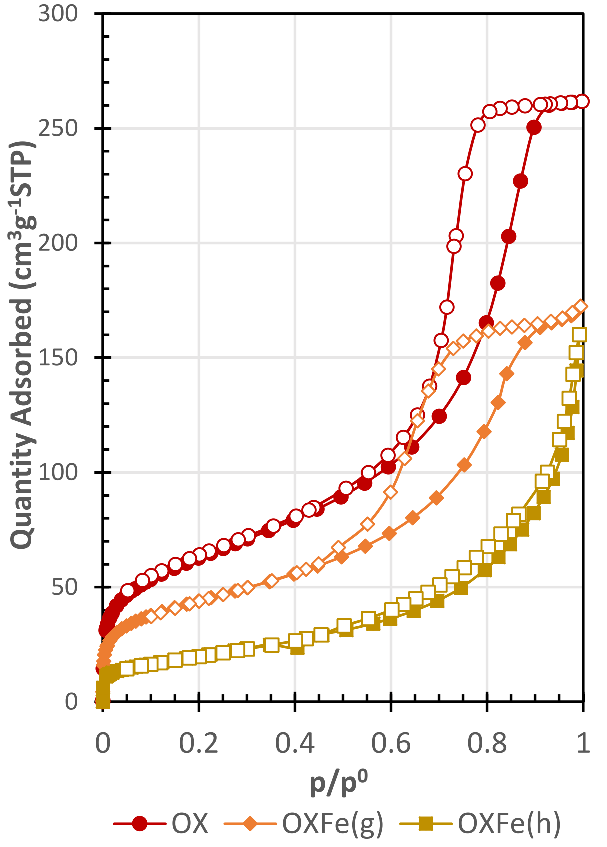

Figures S2. N_2 adsorption-desorption isotherms of the materials OX, OXFe(g), and OXFe(h).

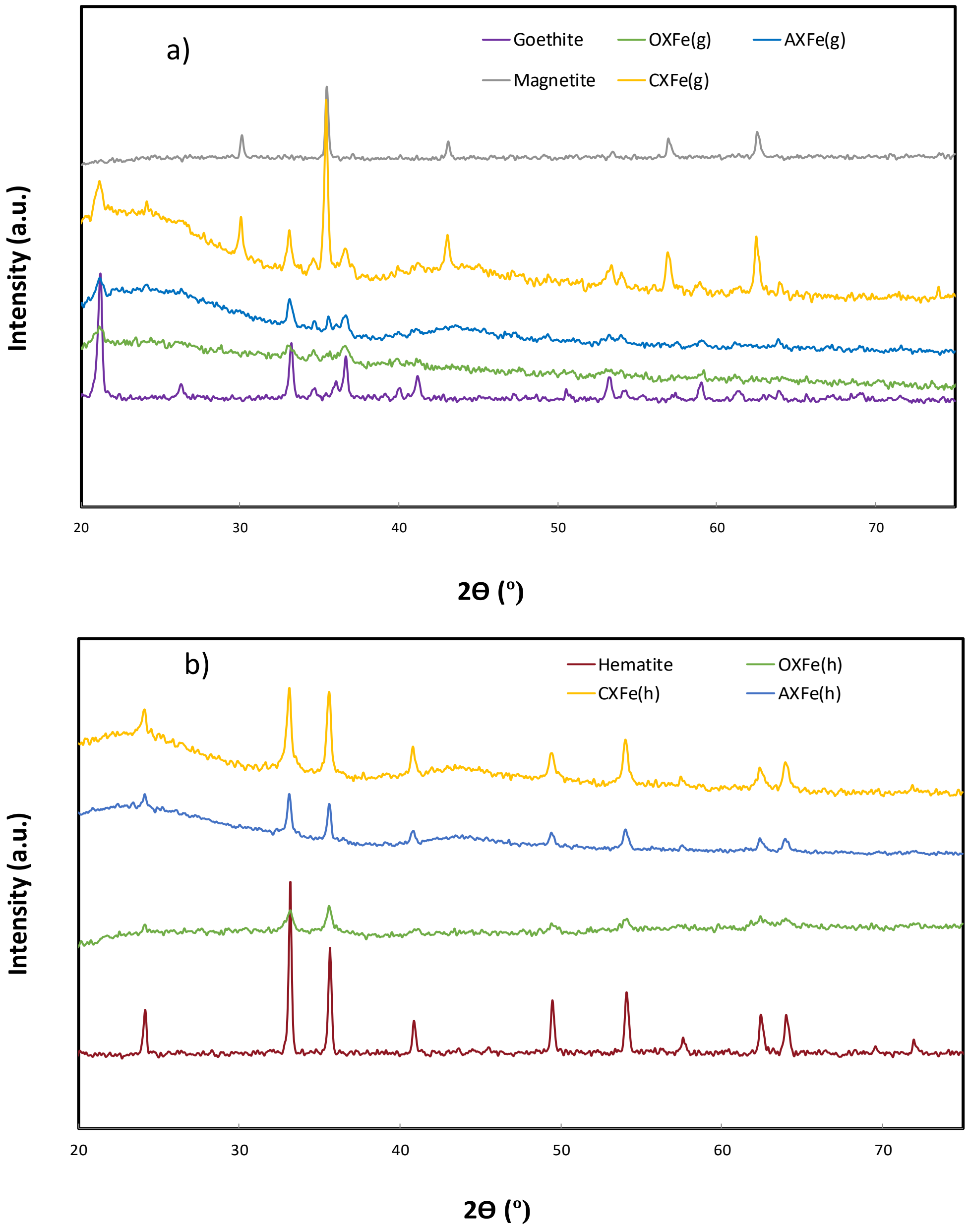

Figure S3. XRD pattern of the carbon materials impregnated with iron species: a) goethite, and b) hematite.

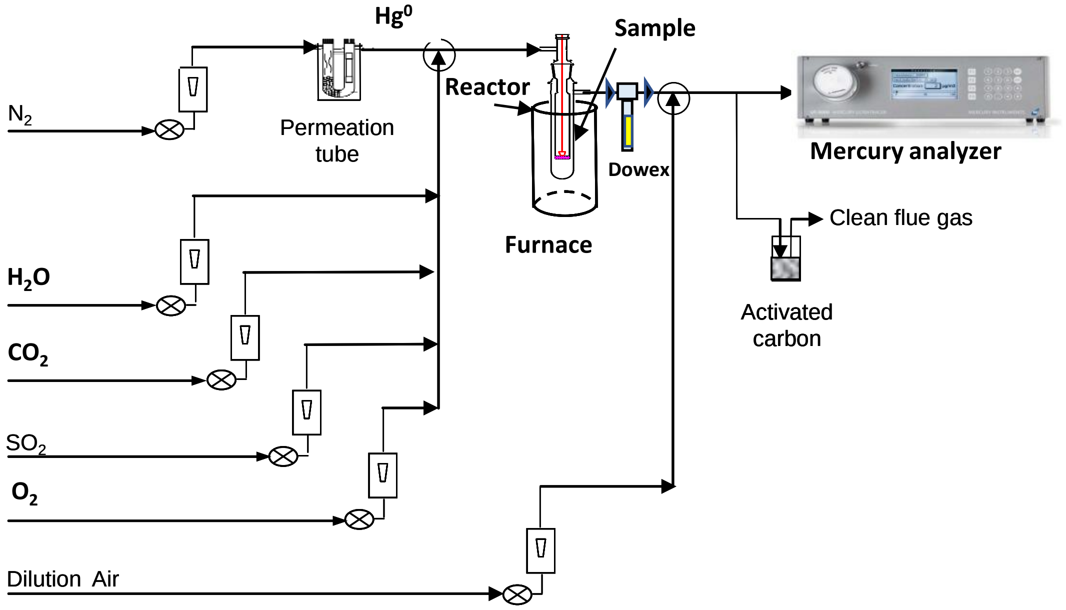

Figure S4. Schematic diagram of experimental device for mercury retention.
